# Editorial: Crosstalk between peripheral and local immune response in the pathophysiology of stroke and neurodegeneration diseases, volume II

**DOI:** 10.3389/fncel.2024.1384276

**Published:** 2024-02-28

**Authors:** Yuanjian Fang, Anwen Shao, Lei Huang, Xiangsheng Zhang, Dirk M. Hermann

**Affiliations:** ^1^Department of Neurosurgery, The Second Affiliated Hospital, School of Medicine, Zhejiang University, Hangzhou, Zhejiang, China; ^2^Department of Neurosurgery, Loma Linda University, Loma Linda, CA, United States; ^3^Department of Physiology and Pharmacology, Loma Linda University, Loma Linda, CA, United States; ^4^Department of Neurosurgery, Beijing Friendship Hospital, Capital Medical University, Beijing, China; ^5^Department of Neurology, University Hospital Essen, University of Duisburg-Essen, Essen, Germany

**Keywords:** immune response, review, original article, stroke, neurodegenerative diseases

The last Research Topic “*Crosstalk between peripheral and local immune response in the pathophysiology of stroke and neurodegeneration diseases*” published in Frontiers in Cellular Neuroscience (Fang et al., 2023) featured 29 papers focusing on peripheral and local immune response in the pathophysiology of stroke and neurodegenerative diseases. The Research Topic covered aspects including pathomechanisms, experimental treatments, and clinical management (Fang et al., 2023). In this volume, nine additional manuscripts were published, including six reviews and three original articles. These new publications primarily focused on advances in mechanisms of immune responses in stroke and neurodegenerative diseases, along with targeted therapies for these diseases.

Systemic and local immune responses play a key role in the development of stroke (Fang et al., 2020; Kelly et al., 2021). Gong et al. comprehensively summarized the role and the interaction between immune system, tissue inflammation, and cell death in ischemic stroke, encompassing the underlying mechanisms and signal pathways. The functions and signal pathways of immune cells, including microglia, astrocytes, neutrophils, T lymphocytes, and monocytes/macrophages, in the post-ischemic brain inflammatory response were discussed. The signal pathways that mediate programmed cell death including pyroptosis, apoptosis, necroptosis, ferroptosis, and PANoptosis were discussed. Mechanisms of action of natural compounds, including salidroside, baicalin, astragaloside IV, and curcumin, in the treatment of ischemic stroke were reviewed, providing potential future directions for ischemic stroke treatment. Deng et al. summarized the latest works on programmed cell death and ferroptosis in subarachnoid hemorrhage (SAH), focussing on iron metabolism, lipid metabolism, antioxidant systems belonging to the GSH/GPX4 system, newly discovered GSH/GPX4-independent antioxidant systems, and their related upstream regulators and downstream targets in the context of early brain injury after SAH.

Peripheral T cells lymphocytes are widely reported to be involved in brain homeostasis as well as neurological diseases (Evans et al., 2019). CD8^+^ T cells are an important population of T cell lymphocytes. Accumulating evidence revealed the roles of CD8+ T cells in acute brain injury in slowly progressive and neurodegenerative diseases. Zhang et al. reviewed the involvement of CD8+ T cells in the regulation of brain injury including stroke, traumatic brain injury, and neurodegenerative diseases, such as Alzheimer's disease and Parkinson's disease. Understanding these processes will promote the investigation of T cell immunity in brain disorders, and provide new intervention strategies for the treatment of brain injuries.

Atherosclerosis, a common cardiovascular disease, is characterized by the dysregulated expression of multiple factors and genes influenced by both environmental and genetic factors. Atherosclerosis is highly correlated with the incidence of ischemic stroke (Tuttolomondo et al., 2020). It has been widely recognized that immune cell infiltration and the interaction of cytokines and chemokines released by these cells contribute to atherosclerotic plaque formation, progression, and regression. Zhao et al. presented a comprehensive overview of the metabolic alterations associated with atherosclerosis, elucidated the impact of inflammatory responses on atherosclerotic plaques, and explored the underlying mechanisms by which statins contribute to plaque stabilization. Furthermore, they investigated the synergistic effects of statins in combination with other pharmacological agents for managing atherosclerosis.

In addition to the reviews concerning the cellular and molecular mechanisms underlying the pathophysiological processes of immune responses in brain injury and neurodegenerative diseases, Sheng et al. and Dong et al. provided a comprehensive summary of the clinical applications of neuroinflammatory molecular imaging and repetitive transcranial magnetic stimulation (rTMS) in brain malignancies and stroke rehabilitation. rTMS, as a novel treatment modulating neural excitability in specific brain regions, has shown promising results in improving post-stroke neurofunction (Starosta et al., 2022). Sheng et al. reviewed the clinical benefits of rTMS for stroke rehabilitation, including enhancements in motor impairment, dysphagia, depression, cognitive function, and central post-stroke pain. They also discussed the underlying molecular and cellular mechanisms involved in rTMS-mediated stroke rehabilitation, particularly focusing on immune regulatory mechanisms such as modulation of immune cells and inflammatory cytokines. Furthermore, they highlighted the current challenges faced by rTMS-mediated stroke rehabilitation along with its future prospects to promote widespread clinical implementation.

Sheng et al. emphasized that incorporating neuroimaging techniques into rTMS-mediated stroke rehabilitation protocols could provide valuable insights into the underlying mechanisms responsible for its effects. Similarly, Dong et al. pointed out that positron emission tomography and magnetic resonance imaging play a crucial role in diagnosing and evaluating brain tumors and associated immune responses. Differentiating between brain tumors and necrotic lesions or inflamed tissues remains a significant challenge in the clinical diagnosis and immunotherapy of brain tumors, which emphasizes the importance of clinically applicable imaging measures monitoring neuroinflammation. They also summarized recent advances in neuroimaging methods aimed at enhancing the specificity of brain tumor diagnosis and evaluating inflamed lesions, which may facilitate the development of non-invasive prognostic and predictive imaging strategies in clinical practice.

Regarding the original articles, Huang et al. investigated the association between the biomarker pan-immune-inflammation value (PIV), which is also called the aggregate index of systemic inflammation (AISI), and all-cause mortality in non-traumatic SAH patients. PIV is calculated by multiplying the counts of neutrophils, monocytes, and platelets, followed by dividing the results by the lymphocyte count. Previous research has demonstrated that PIV serves as a prognostic biomarker for overall survival and progression-free survival in cancer or COVID-19 patients (Yang et al., 2022). The study by Huang et al. included 774 non-traumatic SAH patients, revealing that an elevated PIV upon admission was associated with increased all-cause mortality at various stages (ICU, in-hospital, 30-day, 90-day, and 1-year mortality). These findings emphasize the significance of inflammation-based biomarkers in non-traumatic SAH, and support the predictive value of PIV for predicting outcomes in these patients.

Thougaard et al. isolated peripheral myeloid cells from mice exposed to experimental autoimmune encephalomyelitis (EAE), a multiple sclerosis model, and permanent middle cerebral artery occlusion (pMCAO), a model of ischemic stroke, at different disease time-points, and probed their ability to change the phenotype of primary microglia. They identified peripheral myeloid cell-induced changes in microglia not only dependent on the disease model, but also on the disease phase at which myeloid cells were isolated. Peripheral myeloid cells from acute EAE induced morphological changes in microglia, followed by increases in the expression of genes involved in inflammatory signaling. Conversely, peripheral myeloid cells from the chronic phase of pMCAO induced expression changes in genes involved in inflammatory signaling and phagocytosis, which was not associated with a change in microglia morphology. These finding indicated that neuroprotective and neuroreparative therapies must be tailored to each condition, and no myeloid modulating approach fits all.

Besides, Wang et al. revealed that takinib inhibits microglial M1 polarization and oxidative damage after SAH by targeting the transforming growth factor-β-activated kinase 1 (TAK1)-dependent nod-like receptor pyrin domain-containing protein 3 (NLRP3) inflammasome signaling pathway. They demonstrated that takinib administration significantly inhibited phosphorylated TAK1 expression and promoted M2 microglial polarization. Blockade of TAK1 by takinib reduced neuroinflammation, oxidative damage, brain edema, and neuronal apoptosis, as well as improved neurological deficits after SAH. Moreover, TAK1 also mitigated reactive oxygen species (ROS) production and ROS-mediated NLRP3 inflammasome activation. In contrast, NLRP3 activation by nigericin abated the neuroprotective effects of takinib after SAH. Their findings highlighted that inhibition of TAK1 might be a promising option in the management of SAH.

These studies enriched our understanding of the immune responses in the pathophysiology of stroke and neurodegenerative diseases. We thank all contributing authors, reviewers, and editors who participated in this Research Topic.

## Author contributions

YF: Funding acquisition, Writing—original draft, Writing—review & editing. AS: Writing—review & editing. LH: Writing—review & editing. XZ: Writing—review & editing. DH: Writing—review & editing.
